# Measuring financial protection for health in families with chronic conditions in Rural China

**DOI:** 10.1186/1471-2458-12-988

**Published:** 2012-11-16

**Authors:** Chunhong Jiang, Jingdong Ma, Xiang Zhang, Wujin Luo

**Affiliations:** 1 School of Medicine and Health Management, Huazhong University of Science and Technology, Hang Kong Road, Wuhan, China

**Keywords:** Financial protection, Chronic disease, Rural areas, Poverty, China

## Abstract

**Background:**

As the world’s largest developing country, China has entered into the epidemiological phase characterized by high life expectancy and high morbidity and mortality from chronic diseases. Cardiovascular diseases, chronic obstructive pulmonary diseases, and malignant tumors have become the leading causes of death since the 1990s. Constant payments for maintaining the health status of a family member who has chronic diseases could exhaust household resources, undermining fiscal support for other necessities and eventually resulting in poverty. The purpose of this study is to probe to what degree health expenditure for chronic diseases can impoverish rural families and whether the New Cooperative Medical Scheme can effectively protect families with chronic patients against catastrophic health expenditures.

**Methods:**

We used data from the 4^th^ National Health Services Survey conducted in July 2008 in China. The rural sample we included in the analysis comprised 39,054 households. We used both households suffering from medical impoverishment and households with catastrophic health expenditures to compare the financial protection for families having a chronic patient with different insurance coverage statuses. We used a logistic regression model to estimate the impact of different benefit packages on health financial protection for families having a chronic patient.

**Results:**

An additional 10.53% of the families with a chronic patient were impoverished because of healthcare expenditure, which is more than twice the proportion in families without a chronic patient. There is a higher catastrophic health expenditure incidence in the families with a chronic patient. The results of logistic regression show that simply adding extra benefits did not reduce the financial risks.

**Conclusions:**

There is a lack of effective financial protection for healthcare expenditures for families with a chronic patient in rural China, even though there is a high coverage rate with the New Cooperative Medical Schemes. Given the coming universal coverage by the New Cooperative Medical Scheme and the increasing central government funds in the risk pool, effective financial protection for families should be possible through systematic reform of both financing mechanisms and payment methods.

## Background

Farmers in low income countries live in a risky world. Like drought, flood, and fluctuations in the produce markets, ill health is identified as a major cause of impoverishment in the rural societies of developing countries. There is much literature that documents the vicious circle of disease and poverty [1-7]. As a response to this evidence, there are an increasing number of developing countries that have started health protection programs for their people. Because of limited financial budgets, these health programs largely focus on protecting against catastrophic episodes, for example, hospitalizations inducing large medical expenditures in a short term. However, for those who earn their livings based on their own labor and who have limited household resources, it can also be devastating to encounter chronic conditions without effective financial protections. The constant payments for maintaining the health status of a family member who has chronic diseases can exhaust household resources and eventually result in poverty [8-17]. In the better-off developing countries, the shifting epidemiological phase characterized by increases both in life expectancy and prevalence rates of chronic diseases may pose great challenges to the existing health protection system. In China, cardiovascular diseases, chronic obstructive pulmonary diseases, and malignant tumors have become the leading causes of death for both urban and rural populations since the 1990s. The 4^th^ National Health Service Survey (NHSS) in 2008 documented a 17.1% prevalence rate of chronic diseases in the sample populations of rural areas, which is 4.94% higher than as reported in 2003 based on the 3^rd^ NHSS data. Such a rapid change in disease spectrum highlights the need for policymakers to reshape the health protection system already in place. After the collapse of the traditional health protection system in rural society, the majority of Chinese farmers were exposed unprotected to the uncertainty of medical care for about two decades. Medical spending increased the number of rural households living below the poverty line by 44%. It was not until the year 2003 that the Chinese government launched a new health protection plan for farmers - the New Cooperative Medical Scheme (NCMS), a voluntary medical insurance plan financed by the enrolled families, local governments and the central government. This plan drastically extended coverage to more than 90% of the rural population and was expected to achieve universal coverage by the end of 2010. Though subsidized heavily by the central government, the NCMS does not have a one-size-fits-all detailed plan for implementation across the country except for a couple of principles. Operated by county administrations, the NCMS benefit package and payment methods vary from area to area [18-21]. Such institutional arrangements may offer flexibility in resource allocation and the opportunity for management innovation. Originally, the NCMS was designed to protect against catastrophic health spending, which meant it only covered for inpatient care. To meet the increasing need for chronic care and mediate the consequent economic impact to families, many NCMSs began to incorporate outpatient care insurance or combine it with a medical savings account. Some areas even offer a special reimbursement for patients having chronic diseases from certain conditions. However, we would argue that, because of the lack of high-quality administrative human resources at the county level, such arrangements would only be a result of goodwill rather than scientific, evidence-based policymaking. Based on the data of the latest National Health Services Survey in China, we will answer two questions in this paper: 1) to what degree health spending as a result of the existence of chronic diseases in families can impoverish them; and 2) whether the NCMS can effectively protect families with chronic patients against catastrophic health expenditures.

## Methods

### Data source

We used data from the 4^th^ National Health Services Survey conducted in July 2008 in China. The National Health Services Survey is a cross-sectional survey organized by the Centre for Health Statistics and Information of the Ministry of Health in China. The survey sample adopted multi‐stage stratified random sampling procedures and methods so that it could achieve maximum representation of the demographic and socioeconomic characteristics of the whole population. The rural sample that we included in this analysis comprised 39, 054 households; 13, 990 of them had at least one chronic patient. Data used in this analysis was permitted by China’s Ministry of Health Statistical Information Center.

### Definition of a chronic patient

A chronic patient is someone who was reported to have diabetes, hypertension, heart disease, malignant tumor, or chronic obstructive pulmonary disease diagnosed by doctors in the half year before the survey date. There are certain reasons why we confined chronic illnesses to these five disease clusters. First, they are the leading causes of death and the top prevalent diseases in the rural population in China. Second, we did not focus on one specific disease because many patients actually had one or more chronic co-morbidities. Third, focusing on the five major chronic diseases could reduce the heterogeneity of financial outcomes pertaining to health status, which may vary considerably from disease to disease.

### Measuring financial catastrophe

Following the methodology used by the WHO healthcare financing program [22-25], we used two indicators to estimate financial protection for families with chronic patients: one was to calculate households below the poverty line after healthcare payments out of pocket; the other was to calculate the incidence of catastrophic health expenditure.

1) Defining the poverty line and household subsistence expenditure

A nondiscretionary amount of household financial budget needs to be allocated to basic sustenance in a society. We considered such nondiscretionary spending as the household subsistence expenditure. In our study, we used a food-based poverty line as the household subsistence expenditure. First, we identified households with food expenditure shares of the total household expenditure between the 45^th^ and 55^th^ percentiles. Then, we used equation (1) to calculate the household subsistence expenditure (*hse*).

(1) $$hse=\frac{\sum_{i=1}^{N}\frac{hf}{hs^{\beta }}}{N}hs^{\beta }$$

where *hf* denotes household food expenditure; *hs* denotes household size; *β* is a coefficient for adjusting household sizes; and *N* is the number of households we identified. In this study, *β* was set as 0.56 according to an estimation based on a multi-countries analysis.

2) Defining household capacity to pay

Household capacity to pay reflects the freedom to allocate resources to consumptions beyond subsistence spending. We defined household capacity to pay as the total household expenditure net of subsistence spending. In some cases, households may report food expenditures less than subsistence spending. For such cases, we defined household capacity to pay as the total household expenditure net of food spending. Then, the *i*^th^ household’s capacity to pay (c_*i*_) can be calculated via equation (2)

(2) $$\left\{ \begin{array}{c} c_{i}=he_{i}-hse\;\text{if}\;hse\leq hf_{i}\\ c_{i}=he_{i}-hf_{i}\;\text{if}\;hse>hf_{i} \end{array}\text{,}\right.$$

where *he*_*i*_ denotes the *i*^th^ household’s total expenditure; *hf*_*i*_ denotes the *i*th household’s food expenditure.

3) Defining medical impoverishment

Medical impoverishment refers to non-poor households becoming poor because of out-of-pocket health spending. We defined an indicator, t, which equals 1 when total household expenditure is equal to or larger than subsistence spending but household expenditure minus out-of-pocket health payment is smaller than subsistence spending, and 0 otherwise. Medical impoverishment households (*mih*) are calculated via Equation (3).

(3) $$mih=\sum_{i=1}^{N}t_{i}$$

where N is the sample size.

4) Defining catastrophic health expenditure

Catastrophic health expenditure occurs when out-of-pocket health spending exceeds the threshold fraction of the household’s capacity to pay. We used 0.2 and 0.4 as threshold fractions in our study, but will only report the results based on 0.4. We defined an indicator p to denote the incidence of catastrophic health expenditure. Catastrophic health expenditure households (*che*) are calculated via equation (4).

(4) che=∑i=1Npi,{pi=1ifoopici≥0.4pi=0ifoopici<0.4,

where *oop*_*i*_ is the out-of-pocket health payment of the *i*^th^ household.

### Comparing the different types of insurance coverage

In our dataset, there were 842 households with a chronic patient not covered by any kind of NCMS. For those who were covered by NCMSs, in spite of diversities, their insurance coverage could be divided into 3 groups according to different benefit package designs. We summarize the features and proportions of these 4 kinds of insurance coverage in Table 1. In the CATA group, the insured are reimbursed only for hospitalization episodes, which is essentially catastrophic medical insurance. In the CATAplusA group, besides reimbursement for hospitalization episodes, the insured get reimbursement for out-patient care. But in this case, there is usually a fairly low ceiling on the total reimbursement a household can get. More than half of the sample households were in the CATAplusB group, in which the insured can get extra chronic care compensation when they have certain chronic diseases identified by the authorities, besides the benefit package in the CATAplusA group. The chronic care compensation is limited and covers only a small fraction of the total health spending of the eligible patients. We used both counts of households with medical impoverishment and of households with catastrophic health expenditures to compare the financial protection for families having a chronic patient with different insurance coverage statuses.

**Table 1:** Features and proportion of 4 insurance coverage types in sample areas in China, 2008

**Insurance coverage**	**Features**	**Frequency**	**Percentage**
Uncovered	Not covered by any kinds of NCMSs	2,696	6.90%
CATA	Catastrophic medical insurance	3,439	8.81%
CATAplusA	Catastrophic medical insurance for inpatient care plus compensation for outpatient care	10,720	27.45%
CATAplusB	Catastrophic medical insurance for inpatient care plus reimbursement for outpatient care; limited chronic care compensation for certain chronic diseases	22,199	56.84%

We used a logistic regression model to estimate the impact of different benefit packages on health financial protection for families having a chronic patient.

(5) $$\text{ln}\left(\frac{\text{Pr}\left(\text{y}=1|z\right)}{1-\text{Pr}\;\left(\text{y=}1|z\right)}\right)=\delta z+\beta X+\alpha$$

where *y* is a dummy variable on catastrophic health expenditure (CHE) (1, with CHE; 0 without CHE); *z* is a dummy variable on family’s insurance coverage statuses (3, with CATAplusB; 2, with CATAplusA; 1, with CATA; 0, uncovered) and δ is the coefficient of *z*; *X* is a vector of controlling variables including household income, areas, household size, household head’s education and gender, etc.; β is a vector of parameters for *X*; α is a constant.

## Results

According to our calculation based on the full rural household sample, subsistence expenditure per capita or the poverty line was 2432.64 *yuan* (approx. 355.13 USD in 2008). With this boundary line, 14.25% of the families with chronic patients were poor, compared with 15.63% of the families without chronic patients. However, after paying for health costs, 24.78% of the families with chronic patients were poor, compared with 20.79% of the families without chronic patients. For those who were not poor before health payments, the medical impoverishment incidence rate was much higher among families with a chronic patient than the rate among families without a chronic patient. Table 2 also shows that an additional 10.53% of the families with a chronic patient were impoverished because of paying for health costs, which was more than twice the proportion increase of the families without a chronic patient.

**Table 2:** Effects of health spending on impoverishment in rural China, 2008

	**Total families sampled**	**Pre-payment poverty measure (%)**	**Post-payment poverty measure (%)**	**Percentage point change (%)**
Families with chronic patients	13,990	14.25	24.78	10.53
Families without chronic patients	25,025	15.63	20.79	5.16

**Figure 1:**
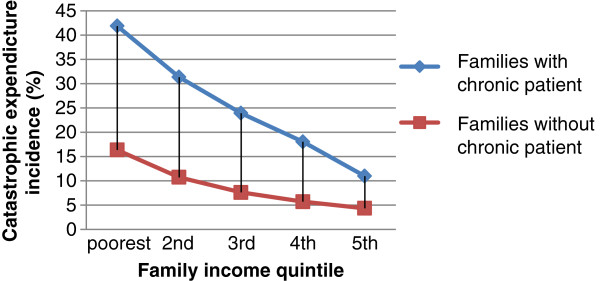
Catastrophic health expenditure incidence by family income quintile in rural China,2008.

**Figure 2:**
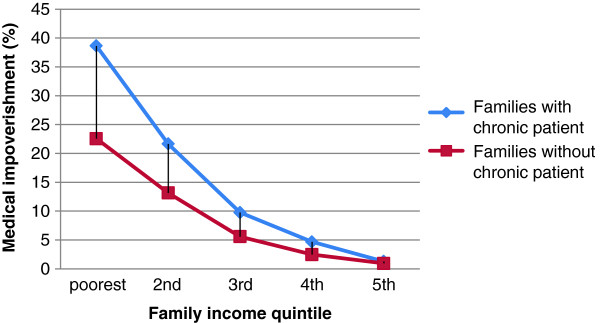
Medical impoverishment by family income quintile in rural China,2008.

There was a higher catastrophic health expenditure incidence in the families with a chronic patient (Figure 1). The catastrophic health expenditure incidence rate varied from 41.86% in the poorest quintile of families with a chronic patient to 10.98% in the richest. The catastrophic health expenditure incidence rates in all families follow a highly regressive distribution. But the pattern in the families with a chronic patient was more serious. Figure 2 shows the medical impoverishment rate in those who were not poor before health payment. The existence of a chronic patient increased the risk of being impoverished in all income quintiles. The distribution of medical impoverishment was fairly regressive in terms of family income. Among families with a chronic patient in the lowest income quintile, the medical impoverishment prevalence was as high as 38.63%. But only 1.28% of families with a chronic patient in the highest income quintile were impoverished because of health spending. Higher income lessened the gap of medical impoverishment between families with and without a chronic patient. Obviously, the existence of a chronic patient induced larger expenditures on health among families in rural China. Although 94% of those families with a chronic patient in our sample were covered by NCMSs, the medical impoverishment and catastrophic health expenditure incidence were still high. Can NCMS coverage and different benefit designs make a difference for the families with chronic conditions in terms of financial protection? Table 3 shows some counterintuitive results. In terms of poverty measurements, the lowest percentage change after payment for health expenses was in those who were not covered by any kind of NCMS. This result could be attributed to adverse selection in the NCMSs since the schemes are voluntary. However, in the other three NCMSgroups, CATAplusA and CATAplusB offered weaker financial protection than did CATA, although those 2 groups were intended to meet the needs of chronically ill patients. It is worth noticing that the families only had the choice to enroll or not, but no choice of different benefit packages once they were enrolled. Table 4 illustrates the medical impoverishment by income quintile under the different insurance coverage statuses. On average, the weakest group for financial protection was CATAplusA, in which 14.18% of the non-poor families became poor after health care payments. But for the lowest income quintile, the weakest group for financial protection was CATAplusB, in which 43.99% of the non-poor families became poor after health care payments. On the contrary, CATA provided the weakest financial protection for families in the highest income quintile. Table 5 shows the results of logistic regression. After controlling for household size, income, gender of household heads, education level of household heads, family member self-perceived illness in the previous 14 days, hospitalization episodes in the previous 1 year, and member clinical visits in the previous 14 days, families in the CATAplusB group still had excess risk (OR: 1.342; 95%CI: 1.105, 1.631) for encountering catastrophic health expenditures compared with families uncovered by NCMSs. CATA offered significant financial protection against catastrophic health expenditures compared with the uncovered group (OR: 0.748; 95%CI: 0.575, 0.972). And CATAplusA had slightly negative impacts on protecting families with a chronic patient from catastrophic health expenditure (OR: 1.174; 95%CI: 0.957, 1.440), although not statistically significantly.

**Table 3:** Effects of NCMS on financial protection for families with chronic conditions

	**Total families sampled**	**Pre-payment poverty measure (%)**	**Post-payment poverty measure (%)**	**Percentage point change (%)**
Uncovered	842	13.78	21.26	7.48
CATA	1,135	22.47	31.81	9.34
CATAplusA	3,661	17.62	29.17	11.55
CATAplusB	8,352	11.70	22.25	10.55
Total	13,990	14.25	24.78	10.53

**Table 4:** Medical impoverishment rate (%) by income quintiles under different insurance coverages in rural China, 2008

	**Family income (*yuan*)**	**uncovered**	**CATA**	**CATAplusA**	**CATAplusB**
lowest	<6500	37.21	41.01	41.49	43.99
2nd	>=6500	17.57	24.29	26.49	19.92
3rd	>=10000	7.73	9.33	9.46	9.13
4th	>=15000	1.79	4.39	4.52	4.59
5th	>=23000	0.46	1.14	1.07	0.93
Total	-	8.68	13.01	14.18	11.79

**Table 5:** Effects of different insurance coverage on catastrophic health expenditure for families with chronic conditions

	**OR**	**Std. Err.**	**Z**	**P**	**[95% Conf. Interval]**	
Household size	0.831	0.015	−10.480	0.000	0.803	0.860
Household income	0.999	0.000	−8.660	0.000	0.999	0.99
Insurance coverage	Reference group: uncovered					
CATA	0.748	0.100	−2.170	0.030	0.575	0.972
CATAplusA	1.174	0.122	1.540	0.123	0.957	1.440
CATAplusB	1.342	0.133	2.960	0.003	1.105	1.631
household head Gender	0.973	0.064	−0.410	0.680	0.856	1.106
Education level of Household head	reference group :never went to school					
Primary school	0.849	0.050	−2.770	0.006	0.756	0.953
middle school	0.677	0.046	−5.750	0.000	0.593	0.774
College or above	0.575	0.060	−5.280	0.000	0.469	0.706
member's self perceived illness in 14 days	1.002	0.058	0.030	0.976	0.895	1.121
Hospitalization Episode	4.155	0.202	29.340	0.000	3.777	4.569
Located areas	reference group : western China					
central China	0.904	0.054	−1.680	0.094	0.804	1.017
eastern China	1.031	0.064	0.500	0.617	0.914	1.164
member's clinic visit in 14 days	1.521	0.077	8.300	0.000	1.378	1.679

## Discussion

The prevalence of chronic diseases in rural China is quite high now. And the figure is projected to be higher in future because of the accelerating population aging process induced by the one –child policy in China. Chronic diseases not only have severely negative impacts on patients’ quality of life, but also cause great losses of welfare to their families. Although the economic consequences of chronic diseases for a family are beyond the direct medical costs, sufficient financial protection against constant outlays for maintaining the health status of a chronic patient is critical for families to avoid poverty or to rebound from it. In our sample, more than 1/3 of the families had at least one chronic patient. Our results show that those families were at higher risk to be impoverished or experience catastrophic health expenditure than the families without a chronic patient. A lower pre-payment poverty rate among families with a chronic patient indicates that they would have been better off if there had been no chronic conditions. Health spending reversed the distribution of poverty between families with or without a chronic patient. For both the families with and without a chronic patient in rural China, the distribution of medical impoverishment and catastrophic health expenditure were regressive in terms of family income. However, such a regressive distribution pattern was much more serious in families affected by chronic diseases. The non-poor families with a chronic patient in the lowest family income quintile were 30 times more likely than those in the highest family income quintile to be impoverished because of health spending. Those who had less family income were more likely to be afflicted by catastrophic health expenditures. Considering the long term effects of chronic diseases, such a regressive distribution indicates that the poorer families with chronic conditions would face a dilemma to either let the health spending squeeze out subsistence spending or to restrict their chronic patient from access to essential healthcare, both of which eventually lead to devastating impacts on livelihood security. Given that more than 94% of the sampled families were covered by the New Cooperative Medical Schemes, these should have offered a certain financial protection for the families with a chronic patient. However, our results show that the families covered by NCMSs were at higher risk to be impoverished than the uncovered. Given the possibility of adverse selection in the voluntary insurance schemes, or that some of the uncovered may be rich enough to self-insure, the credibility of this result may be undermined by these potential selection biases. Nonetheless, this is consistent with other reports of the impact of NCMS [26-30]. For instance, Wagstaff and Lindelow found that health insurance in China increases the risk of high and catastrophic spending using probit regression models based on the data of China Health and Nutrition Survey. Wagstaff and Lindelow reported in another study that the NCMS had not reduced out‐of‐pocket spending for either outpatient or inpatient visits. Our results also indicate that simply adding extra benefit packages to catastrophic medical insurance does not necessarily result in more effective financial protection. Winnie Yip and William Hsiao argued that the weak performance of NCMSs in financial protection could be largely attributed to ignorance of the high prevalence of chronic diseases and of the corresponding medical expenditure pattern in policy design. Adding extra insurance for outpatient care or compensation for chronic care of certain diseases should have been a response to such arguments. But these decisions were largely made by administrators at a county level, who are in lack of specific knowledge in designing health financing system. The corresponding policy change could not be delivered in a systematic way. Without consonance of payment reform and effective disease management, the effect of an extra benefit package for chronic patients will be decoupled by moral hazard or other sequent behavior changes on both supply and demand sides. There are several limitations of our study in terms of policy implications. First, although our study has roughly shed light on how different benefit package designs potentially affect financial protection for families with a chronic patient in rural China, methodologically it is not a strict evaluation of the effectiveness of the new cooperative medical schemes. There are likely to be selection biases in our study. However, this limitation may be mitigated to some extent by the high enrollment rate in our sample. Second, our dataset does not contain information on the behaviors of patients and physicians. We cannot provide empirical evidence that behavior changes lead to overuse and cost inflation which could explain the excess financial risk in more generous benefit packages. Third, our data come from a cross-sectional survey so that we cannot take into account poverty dynamics caused by the long-term effects of chronic diseases.

## Conclusions

In summary, there is a lack of effective financial health protection for families with a chronic patient in rural China, even though there is a high coverage rate with the New Cooperative Medical Schemes. Driven by the high prevalence of chronic disease and the desire for a better quality of life, the demand for chronic care will be consistently increasing. This would be a great challenge for the effectiveness and sustainability of the rural health insurance system in China. Given the coming universal coverage of NCMS and the increasing central government funds in the risk pool, effective financial protection for families should be provided through systematic reform of both financing mechanisms and payment methods.

## Abbreviations

NCMS: The new cooperative medical scheme.

## Competing interests

The authors declare that they have no competing interests.

## Authors’ contributions

Study concept and design: CJ and JM. Statistical analysis: CJ, JM, XZ and WL.Analysis and interpretation of data: CJ, JM, XZ and WL.Draft of the manuscript: CJ and JM. Critical revision of the manuscript for important intellectual content:JM and CJ.Administrative, technical, and material support: JM and CJ. Study supervision: JM. All authors read and approved the final manuscript.

## Pre-publication history

The pre-publication history for this paper can be accessed here:

http://www.biomedcentral.com/1471-2458/12/988/prepub

## References

[B1] Liu Y Rao K Fei J Economic transition and health transition: comparing China and Russia Health Policy 1998 44 2 103 122 10.1016/S0168-8510(98)00010-4 10180676

[B2] Smith JP Healthy bodies and thick wallets: the dual relation between health and economic status J Econ Perspect 1999 13 2 145 166 10.1257/jep.13.2.145 PMC3697076 15179962

[B3] Dercon S Income risk, coping strategies, and safety nets World Bank Res Obs 2002 17 2 141 166 10.1093/wbro/17.2.141

[B4] Carter MR Maluccio JA Social capital and coping with economic shocks: an analysis of stunting of south African children World Dev 2003 31 7 1147 1163 10.1016/S0305-750X(03)00062-7

[B5] Jensen RT Richter K The health implications of social security failure: evidence from the Russian pension crisis J Public Econ 2004 88 1–2 209 236

[B6] Russell S The economic burden of illness for households in developing countries: a review of studies focusing on malaria, tuberculosis, and human immunodeficiency virus/acquired immunodeficiency syndrome Am J Trop Med Hyg 2004 71 suppl 2 147 155 15331831

[B7] Carter MR Little PD Mogues T Negatu W Shocks, sensitivity and resilience: tracking the economic impacts of environmental disaster on assets in Ethiopia and Honduras 2004 In Staff Paper Series, Madison: University of Wisconsin, Agricultural and Applied Economics 489

[B8] Druss BG Marcus SC Olfson M Tanielian T Elinson L Pincus HA Comparing the national economic burden of five chronic conditions Health Aff 2001 20 6 233 241 10.1377/hlthaff.20.6.233 11816664

[B9] Hwang W Weller W Ireys H Anderson G Out-of-pocket medical spending for care of chronic conditions Health Aff 2001 20 6 267 278 10.1377/hlthaff.20.6.267 11816667

[B10] Joyce GF Keeler EB Shang B Goldman DP The lifetime burden of chronic disease among the elderly Health Aff 2005 24 Suppl 2 W5R18 29 10.1377/hlthaff.w5.r18 PMC6385883 16186148

[B11] World Health Organization and Public Health Agency of Canada Preventing chronic diseases: a vital investment 2005 (Ottawa): World Health Organization; Public Health Agency of Canada, Geneva http://www.who.int/chp/chronic_disease_report/contents/en/index.html

[B12] Piette JD Heisler M Horne R Alexander GC A conceptually based approach to understanding chronically ill patients' responses to medication cost pressures Soc Sci Med 2006 62 4 846 857 10.1016/j.socscimed.2005.06.045 16095789

[B13] Suhrcke M Nugent RA Stuckler D Chronic disease: an economic perspective 2006 Oxford Health Alliance, London

[B14] Abegunde DO Mathers CD Adam T Ortegon M Strong K The burden and costs of chronic diseases in low-income and middle-income countries Lancet 2007 370 9603 1929 1938 10.1016/S0140-6736(07)61696-1 18063029

[B15] Adeyi O Smith O Robles S Public policy and the challenge of chronic noncommunicable diseases 2007 World Bank, Washington, D.C.

[B16] Abegunde DO Stanciole AE The economic impact of chronic diseases: how do households respond to shocks? Evidence from Russia Soc Sci Med 2008 66 11 2296 2307 10.1016/j.socscimed.2008.01.041 18329147

[B17] Stuckler D Population causes and consequences of leading chronic diseases: a comparative analysis of prevailing explanations Milbank Q 2008 86 2 273 326 10.1111/j.1468-0009.2008.00522.x 18522614 PMC2690359

[B18] Feng XS Tang SL Bloom G Segall M Gu XY Cooperative medical schemes in contemporary rural China Soc Sci Med 1995 41 8 1111 1118 10.1016/0277-9536(94)00417-R 8578334

[B19] Lei X Lin W The New cooperative medical scheme in rural China: does more coverage mean more service and better health? Health Econ 2009 18 S2 S25 S46 10.1002/hec.1501 19551752

[B20] You XD Kobayashi Y The new cooperative medical scheme in China Health Policy 2009 91 1 1 9 10.1016/j.healthpol.2008.11.012 19121873

[B21] Zhang L Cheng X Liu X Zhu K Tang S Bogg L Dobberschuetz K Tolhurst R Balancing the funds in the new cooperative medical scheme in rural China: determinants and influencing factors in two provinces Int J Health Plann Manage 2010 25 96 118 19582799 10.1002/hpm.988

[B22] Xu K Evans DB Kawabata K Zeramdini R Klavus J Murray CJL Household catastrophic health expenditure: a multicountry analysis Lancet 2003 362 9378 111 117 10.1016/S0140-6736(03)13861-5 12867110

[B23] Kawabata K Xu K Carrin G Preventing impoverishment through protection against catastrophic health expenditure Bull World Health Organ 2002 80 8 612 612 12219150 PMC2567587

[B24] Xu K Evans DB Kadama P Nabyonga J Ogwal PO Nabukhonzo P Aguilar AM Understanding the impact of eliminating user fees: Utilization and catastrophic health expenditures in Uganda Soc Sci Med 2006 62 4 866 876 10.1016/j.socscimed.2005.07.004 16139936

[B25] Limwattananon ST Angcharoensathien V Prakongsai P Catastrophic and poverty impacts of health payments: results from national household surveys in Thailand Bull World Health Organ 2007 85 8 600 606 10.2471/BLT.06.033720 17768518 PMC2636377

[B26] Yip W Hsiao WC The chinese health system at a crossroads Health Aff 2008 27 2 460 468 10.1377/hlthaff.27.2.460 18332503

[B27] Sun X Jackson S Carmichael G Sleigh AC Catastrophic medical payment and financial protection in rural China: evidence from the New Cooperative Medical Scheme in Shandong Province Health Econ 2009 18 1 103 119 10.1002/hec.1346 18283715

[B28] Wagstaff A Social health insurance reexamined Health Econ 2009 19 503 517 19399789 10.1002/hec.1492

[B29] Wagstaff A Lindelow M Gao J Xu L Qian JC Extending health insurance to the rural population: an impact evaluation of China's new cooperative medical scheme J Health Econ 2009 28 1 1 19 10.1016/j.jhealeco.2008.10.007 19058865

[B30] Yi H Zhang L Singer K Rozelle S Atlas S Health insurance and catastrophic illness: a report on the new cooperative medical system in rural China Health Econ 2009 18 S2 S119 S127 10.1002/hec.1510 19551747

